# Lithium promoted mesoporous manganese oxide catalyzed oxidation of allyl ethers

**DOI:** 10.1038/s41467-019-08619-x

**Published:** 2019-02-08

**Authors:** Biswanath Dutta, Ryan Clarke, Sumathy Raman, Timothy D. Shaffer, Laura Achola, Partha Nandi, Steven L. Suib

**Affiliations:** 1 Department of Chemistry, University of Connecticut, U-3060, 55 North Eagleville Road, Storrs, CT 06269 USA; 2 Corporate Strategic Research, ExxonMobil, 1545 US 22 East, Annandale, NJ 08801 USA; 3 Institute of Materials Science, University of Connecticut, U-3060, 55 North Eagleville Road, Storrs, CT 06269 USA

## Abstract

Herein we report the first example of the catalytic aerobic partial oxidation of allyl ether to its acrylate ester derivative. Many partial oxidations often need an expensive oxidant such as peroxides or other species to drive such reactions. In addition, selective generation of esters using porous catalysts has been elusive. This reaction is catalyzed by a Li ion promoted mesoporous manganese oxide (meso-Mn_2_O_3_) under mild conditions with no precious metals, a reusable heterogeneous catalyst, and easy isolation. This process is very attractive for the oxidation of allyl ethers. We report on the catalytic activity, selectivity, and scope of the reaction. In the best cases presented, almost complete conversion of allyl ether with near complete chemo-selectivity towards acrylate ester derivatives is observed. Based on results from controlled experiments, we propose a possible reaction mechanism for the case in which *N*-hydroxyphthalimide (NHPI) is used in combination with trichloroacetonitrile (CCl_3_CN).

## Abstract (web summary)

Acrylics and acrylates have become important building blocks for the chemical industry, but their efficient synthesis remains a challenge. Here, the authors report the first example of the catalytic aerobic partial oxidation of allyl ether to its acrylate ester derivative using a Li ion promoted mesoporous manganese oxide under mild conditions.

## Introduction

Acrylics and acrylates^1–3^ have become important building blocks for the chemical industry today. Acrylates are key ingredients for producing dyes, glues, papers, adhesives, binders, paints, and thickeners^4,5^. The industrial source of acrylics and acrylates is propylene that is produced as either the by-product of fluid catalytic cracking (FCC)^6^, steam cracking, or by propane dehydrogenation^7^. Propane or propylene can be subsequently oxidized to acrylates via the Mitsubishi process^8^ or propene ammoxidation^9–11^ using mixed metal oxides. These processes involve the formation of acrylonitrile intermediates that are converted into acrylates, acrylamides, and acrylic acids depending on the reaction condition employed^12^. Typically these oxidations are run around 450 °C, where formation of ethylene, HCN, acetonitrile, N_2_O, and CO_2_ by-products are unavoidable.

Recently, several homogeneous processes have been recognized for efficiently oxidizing allylic C–H bonds^13–15^. Peroxides and N-hydroxides were used for synthesizing various enones via allylic C–H oxidation^14^. However, these methodologies primarily focus on cyclic unsaturated molecules. Crystalline manganese oxide based materials (e.g., K-OMS-2) are used for various catalytic transformations including the selective oxidation of alcohols to aldehydes^16^, hydrocarbons to alcohols and ketones^17^, styrenes to styrene oxides^18^, alcohols to amides^19^, amines to imines^20^, some oxidative coupling reactions, such as alkynes to di-ynes^21^, alkyne-silanes^22^, anilines to azobenzenes^23^, and dehydrogenative oxidation reaction of alkanes^24^.

These mixed valent manganese oxide materials have numerous structural forms with high thermal stability^25^. The reduction of Mn^4+/3+^ to Mn^3+/2+^ releases labile lattice oxygen atoms, which are responsible for their catalytic performance^21,25^. These lattice oxygens act as basic sites for H abstraction during the reaction. This eliminates the requirement of using a base with mesoporous manganese oxides for oxidative dehydrogenations^26^. Moreover, the presence of alkali and alkaline earth metal ions in the manganese oxide lattice enhance catalytic activity. Basicity increases due to the attraction of lattice oxygens toward these electropositive ions. We therefore introduced these types of ions in the allylic C–H oxidation reactions. These mesoporous manganese oxide materials, developed by an inverse-micelle method, were selected for their comparatively high surface areas compared with traditional mesoporous transition metal oxides^27^.

In this paper, acrylates have been directly produced via an aerobic catalytic oxidation process. Allyl ethers were used in this low-temperature process with lithium ion impregnated mesoporous manganese oxides as heterogeneous catalysts. An advantage of these catalysts is that manganese oxides are abundant and inexpensive materials. Conversions of diallyl ether as high as 95% with selectivities to allyl acrylate greater than 99% were achieved via optimization of the reaction conditions. Such studies suggest that NHPI–air, hydrogen peroxides, and CCl_3_CN are essential in controlling activity, selectivity, and stability of these catalysts. Control experiments were done to determine the interrelationships of these critical factors in these oxidation reactions. A plausible mechanism is proposed based on mechanistic studies. Such studies suggest that radical intermediate formation, followed by successive hydroxylation and oxidation are significant for this reaction. Mobility of lattice oxygen in the meso-Mn_2_O_3_ catalysts and CCl_3_CN promoted PINO radicals are the significant requirements in this catalytic process.

## Results

### Reaction conditions

The interdependence and synergistic effect of the reaction parameters spurred us to evaluate and compare reaction conditions (Supplementary Figs. 1 and 2). Reaction kinetics under different set of conditions were investigated. NHPI–air–CCl_3_CN emerged with the highest rate constant of 2.82 min^−1^, while NHPI–N_2_–CCl_3_CN displayed the slowest rate constant of 0.3 min^−1^ (Supplementary Table 6, entry 1 and 4). Moreover, reaction under NHPI–air with no CCl_3_CN displayed a rate constant of 0.57 min^−1^, which was slightly lower than 0.71 min^−1^ of the TBHP–CCl_3_CN system (Supplementary Table 6, entry 2 and 3). The fourfold increase in activity for NHPI–air–CCl_3_CN than the TBHP–CCl_3_CN system proved its superiority. A ninefold decrease in activity upon replacing air with nitrogen and a fivefold decrease in the absence of CCl_3_CN, proved their significance. These studies helped evaluate the importance of reaction parameters. According to the results obtained, reaction conditions which increase the reaction rate are ranked as follows: NHPI–O_2_–CCl_3_CN > TBHP–CCl_3_CN > NHPI–O_2_– no CCl_3_CN > NHPI–N_2_–CCl_3_CN. In all cases, these kinetic experiments revealed a first-order rate dependence with respect to the starting material allyl ether. Further characterization was done with X-ray powder diffraction and BET surface area measurements (Supplementary Fig. 6). Various reactants and products were analyzed with nuclear magnetic resonance (NMR), See Supplementary Note 1.

### Substrate scope

The substrate scope and limitations were then explored using structurally different ethers, with each possessing at least one allylic CH_2_ group. Under optimized reaction conditions, diallyl ether and cyclic dihydrofuran (Table 1, entry 1 and 3) were transformed to their corresponding oxidized products exclusively with 92 and 99% yields, respectively. On the other hand, allyl acetate and allyl glycidyl ether (Table 1, entry 2 and 4) were transformed to their oxidized counterpart with 60 and 66% yields, respectively. However, a poor yield of 12% was obtained for allyl phenyl ether (Table 1, entry 5). Hyperconjugation and the loss of a proton from the α-carbon was easier in the case of allyl ether than that of phenyl allyl ether (B. E. = 67.2 ± 0.5, and 81.84 ± 0.5 kcal/ mol, respectively, Table 1). This resulted in better stability and poorer reactivity of the phenyl allyl ether than the allyl ether. To investigate the reason for selective formation of monoesters over the anhydride, density functional theory (DFT) calculations were conducted, using the CBS–QB3 basis set. DFT results show that C–H bond dissociation energy of allylic C–H of diallyl ether is 80.8 kcal/mol, which significantly increases to 82.7 kcal/mol for the allyl acrylate ester C–H. A smaller increase in bond dissociation energy was observed when comparing reactants and products in Table 1, entry 3. Perhaps the cyclic nature of the substrate binding to the catalyst surface further limited the oxidation of the lactone to the corresponding anhydride. In Table 1, entry 4 the glycidyl C–H bond in the reactant is a competing site for oxidation with a calculated BDE of 91.2 kcal/mol. Upon oxidation of the allylic C–H bond the glycidyl C–H bond becomes stronger at 95.7 kcal/mol. Probably for similar reasons, no activation of α-C–H bonds was noted in 1-butene and ethyl substituted allyl ethers (Table 1, entry 6 and 7).

**Table 1:** Substrate scope of the reaction protocol^a^

^a^Reaction condition: substrate (2 mmol), Temp- 80 °C, solvent- 2.5 mL, Catalyst- meso-Li-Mn_2_O_3_ (25 mg), NHPI (25 mol%), 6 h and air balloon. ^b^Bond dissociation energies (BDE) were calculated by DFT using CBS–QB3 basis set. Numbers in parenthesis are the isolated yields. ^c^Conversions and selectivities were determined by GC-MS (in all cases >99% selectivity were obtained). ^d^Yield = Conversion × selectivity (%), ^e^TOF = TON/h, TON = mole of ether converted to the product over mole of catalyst used. ^f^8 h, ^g^16 h

### Stability and reusability

To determine if the observed catalysis is a result of leached active sites, the solid catalyst was separated from the reaction mixture by hot filtration after 45 min (about 25% conversion). The resulting filtrate was then subjected to reaction conditions for an additional 315 min. Aliquots were collected successively after each hour and were analyzed with GC-MS to track the progress of the reaction. No further improvement in GC yield was observed after the first 2 h (Supplementary Fig. 3), indicating no further conversion of substrate in the absence of a catalyst. The same filtrate was also analyzed by inductively coupled plasma mass spectrometry (ICP-MS), which indicated the presence of a very low amount (165 ppm) of Manganese (Mn).

To verify reusability, the catalyst was retrieved (> 90%) after the reaction by simple filtration and washed with excess acetonitrile and methanol. The catalyst was reactivated prior to reuse at 250 °C for 30 min under air to remove any adsorbed substrates. As evident from Supplementary Fig. 4, the catalyst was successfully reused for three more cycles with less than a 50% drop in activity. The decrease in activity is probably due to metal poisoning (Mn^III^), caused by the strong coordination of different radicals with the metal centers. Therefore, our catalyst can be considered as truly heterogeneous, stable, and moderately reusable.

## Discussion

**Fig. 1:**
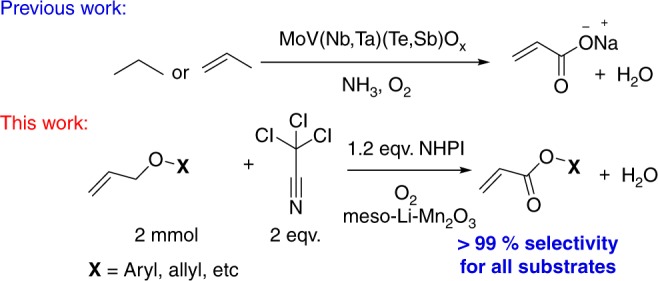
Schematic representation of the plausible reaction mechanism in presence of PINO, MeCN, and O_2_. This mechanistic scheme identifies all of the reaction intermediates, shows the various surface manganese oxide species used in the catalytic reaction, and shows a detailed set of pathways that account for observed products

Based on the above-mentioned observations, we propose mechanisms by both TBHP–CCl_3_CN and NHPI–air–CCl_3_CN catalysts. The role of manganese species during the reaction can be correlated with the work reported by Ishii et al. using electron paramagnetic resonance (EPR) measurements^28^. To further understand our systems, EPR data for the acetonitrile solution of NHPI with and without meso-Mn_2_O_3_ under atmospheric condition were compared. There was a decrease in the EPR signal, which could be due to the formation of an extra radical (possibly PINO) in the former reaction at g = 2.0073 (Supplementary Fig. 5). This suggest the formation of a superoxomanganese (IV) or µ-peroxomanganese (IV) complex between Mn^III^ and O_2_. In both cases, a series of steps contributed to the formation of monoesters from the allyl ethers. Manganese oxides (Mn^IV^–oxide, **1**) have been reported to react with molecular oxygen to form Mn^IV^–peroxide radicals (**2**) (Fig. 1). These are considered to be very reactive and are expected to deprotonate the NHPI molecule to form a PINO radical and a Mn^IV^–hydrogen peroxide (**3**) species. A competition between PINO radical and Mn^IV^–peroxide radical (**2**, is expected during the deprotonation of allylic hydrogens (**5**). The corresponding radical (**6**) is believed to couple with an oxygen molecule and Mn^II^ species to form an intermediate **7** that upon reacting with a molecule of NHPI (**4**) produced a Mn^III^–PINO (**11**) and allyl ether-hydrogen peroxide (**8**) species. The latter went through further rearrangements with the loss of an oxygen moiety to generate the desired allyl acrylate (**10**), whereas the Mn^III^–PINO (**11**) regenerated the TBHP and Mn^II^ species.

**Fig. 2:**
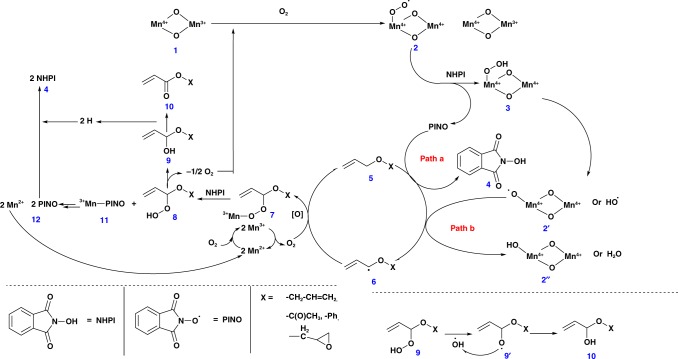
This figure compares earlier mechanisms for acrylate formation using mixed metal oxides, oxygen, and ammonia versus this work using *N*-hydroxyphthalimide (NHPI), oxygen, and lithium manganese oxides which leads to enhanced (99%) selectivity for all substrates^8–11^

The mechanism in the presence of TBHP is expected to follow a similar mechanism to that of the NHPI catalyzed pathway **(**Fig. 2**)**. A fourfold decrease in the rate constant (i.e., from 2.82 min^−1^ for NHPI–O_2_–CCl_3_CN to the 0.71 min^−1^ by TBHP–CCl_3_CN-based reaction) discouraged us from using this catalytic system. Moreover, we have independently determined that Mn_2_O_3_ is an extremely potent material for decomposing TBHP into t-BuOH and O_2_^29^. Since O_2_ is highly abundant and cheap, we focused our work using O_2_ as an oxidant, not only due to the relatively poor rates of TBHP–CCl_3_CN system.

In both cases, probably an intermediate **6 type** radical was oxidized to form an α-hydroxy ether (**9**). The transformation of intermediate **8** to **9**, probably resulted in the release of oxygen, which are the terminal oxidants of the catalytic process (Fig. 1). Though the fate of this oxygen is known, their method of interaction in the oxidation process is still unknown. An α-hydroperoxide intermediate is likely to be formed, which, however, is very difficult to trap in rapid oxidations over manganese oxide^30^.

In summary, we have developed a low-temperature, heterogeneous catalytic aerobic oxidation procedure to form acrylates directly from allyl ethers using lithium ion impregnated mesoporous manganese oxide material. Mn being an earth-abundant element and O_2_ being the most abundant oxidant makes this catalytic reaction attractive. With diallyl ether we achieved a conversion of 95% to allyl acrylate with a selectivity of > 99% after extensive optimization of the reaction conditions. This revealed the crucial role of NHPI–air, hydrogen peroxides, and CCl_3_CN in promoting the activity of the catalyst. The interdependence of these promoting agents along with the catalyst in driving the catalytic process was interpreted by control experiments that led to a plausible mechanism. Mechanistic studies invoked the possibility of radical intermediate formation, followed by successive hydroxylation and oxidation. The presence of the CCl_3_CN promoted PINO radical and labile lattice oxygen of the meso-Mn_2_O_3_ material are the most important features of this catalytic process.

## Methods

### Catalysis

In this paper, we have developed a new catalytic approach to the mild aerobic partial oxidations of diallyl ether, see Fig. 2. Diallyl ether was chosen as a model substrate that could potentially suffer from (i) a double-bond shift and (ii) oxidation of the olefin to aldehyde or epoxide prior to the activation of allylic C–H bond. We demonstrate the aerobic oxidation of allyl ethers to corresponding acrylate esters using various cation doped mesoporous manganese oxide materials. The absence of any precious metal, easy isolation of the products, and the use of a reusable heterogeneous catalyst in the absence of acidic or alkaline media makes this process attractive for the oxidation of allyl ethers.

We initiated the oxidation of allyl ether in air as a model reaction to determine the optimal conditions. In our early screening attempts, we evaluated the impact of solvent on this oxidation. The results of our screening studies are presented in Supplementary Table 1 of the Supplementary information. Interestingly, no trace of the oxidized product was detected until *tert*-butylhydroperoxide (TBHP) was introduced (Supplementary Table 1, entry 1–4). Unsatisfactory conversion of allyl ether, even after using TBHP led us to employ a radical promoter. In 2010, Kamijo et al.^31^ successfully discovered the promotional activity of trichloroacetonitrile (CCl_3_CN) for m-CPBA (metachloroperbenzoic acid) in either oxidations. The high electrophilicity of the CCl_3_CN most likely promoted the homolytic cleavage of O–O bonds of *m*CPBA by forming a highly unstable peroxyimidate adduct. The utilization of this hydroperoxide promoter (CCl_3_CN) as a solvent increased the product formation in our system to as high as 82% (Supplementary Table 1, entry 5). Further optimization of the amount of this promoter (CCl_3_CN) displayed similar results (81% yield, Supplementary Table 1, entry 6), indicating no dependence of reactivity on the amount of CCl_3_CN used. The role of CCl_3_CN was further validated by performing a solvent-free reaction with only 15% of the allyl ether converted (Supplementary Table 1, entry 7). The promising results of solvent optimization led to evaluation of other metal oxide supports. None were found to be as active as meso-Mn_2_O_3_ (81% yield) (Supplementary Table 2, entry 1–4) with at most 20% yield observed for the other supports. Better accessibility of the labile lattice oxygens and comparatively lower binding energy of metal–oxygen bonds resulted in higher yields^20^. Reactions using Mn^3+^-enriched species (such as c-Mn_2_O_3_ and Mn(OAc)_3_) showed better conversions than both the Mn^2+^–rich and catalyst-free systems (Supplementary Table 2, entry 6–9). The homogeneous reaction performed in the presence of a Mn^3+^-enriched (Mn(OAc)_3_) species demonstrated lower yields (only 13%) than its heterogeneous counterpart (c-Mn_2_O_3_), which was probably due to the lack of lattice oxygens in the homogeneous systems. Moreover, a heterogeneous system can adsorb the substrate (allyl ether) prior to oxidation with lattice oxygens, which is not possible in a homogeneous system. The presence of 1 mol% of electropositive Cs^+^ ions in mesoporous manganese oxide increased the oxidative property of the material by 100 times for alcohol to aldehyde and amine to imine formation^20,26^. This spurred us to investigate the effect of alkali and alkaline earth metal ions on the heterogeneous meso-Mn_2_O_3_ system. Nearly all showed an enhancement in activity (Supplementary Table 3). Enhancement of the surface basicity of the material due to the incorporation of alkali and alkaline earth metal ions is a likely reason for this excellent activity. Introduction of these electropositive alkali and alkaline earth metal ions on the surface are potentially known to cause both surface defects and accumulation of negative charges, which enhance the surface basicity^32^. This inspired us to evaluate the effect of different alkali and alkali metal ions. Li impregnated meso-Mn_2_O_3_ (meso-Li-Mn_2_O_3_) emerged as the best catalyst with a yield of 92% (Supplementary Table 3, entry 1) and a selectivity of > 99%. Perhaps, due to the higher charge density of Li^+^ ions, their bonding on the manganese oxide surface was enhanced. Li^+^, the smallest ion, could also favor the introduction of more ions onto the surface of manganese oxide nanoparticles and lead to the best activity. The effect of other peroxides was explored in the presence of a catalyst. Benzoyl peroxide appears to be a suitable alternative to TBHP, converting 90% of the allyl ether (Supplementary Table 4, entry 1–6). Despite these high yields, multiple side products from the peroxides were observed. To minimize these side products*, N*-hydroxyphthalimide (NHPI) was used as it is known for its powerful oxidizing ability in combination with transition metals^13–15,33^. This species converted 95% of the allyl ether to the expected monoester with > 99% selectivity, and fewer side products than before (Supplementary Table 4, entry 7). Consequently, NHPI in air was considered to be the most promising oxidant for this system. Furthermore, adjusting the NHPI amount beyond 25 mol% (Supplementary Table 5) did not show a detectable difference in the performance of the system. After extensive screening (see the supporting information), the best catalytic system was achieved with 1 mol% Li ion on meso-Mn_2_O_3_ catalyst, in the presence of 25 mol% of NHPI, in air with a minimum of two equivalents of CCl_3_CN in acetonitrile at 80 °C (95% conversion and >99% selectivity).

### Mechanistic studies

Once the catalytic system was optimized, additional experiments were performed to further investigate the mechanistic details of the catalytic process. The experiment in the presence of NHPI and metal oxide catalyst was initially performed in the presence of a radical promoter CCl_3_CN. CCl_3_CN is expected to promote the activity of hydroperoxide radicals. A detectable drop in conversion from 95 to 80% was noticed when CCl_3_CN was excluded (Supplementary Table 4, entry 9). This suggests that CCl_3_CN promotes the generation of *N*-hydroxides to form *N*-oxide radicals. Interestingly, only 6% of the product formation was detected when air or oxygen was replaced by nitrogen in the presence of NHPI (Supplementary Table 4, entry 10). This confirmed the role of air in promoting formation of the PINO radical from NHPI. In addition, this small but significant activity under a nitrogen atmosphere (18% conversion) implied that the aerobic oxygens were not the terminal oxidants. An oxidant which causes the termination of a catalytic process is called the terminal oxidant. The nitrogen atmosphere affected the reaction by slowing down the kinetics of PINO formation. Evaluating the terminal oxidant for an oxidation reaction is essential in controlling kinetics. To further probe the mechanism, the CCl_3_CN promoter was excluded from a reaction conducted in a nitrogen atmosphere and the conversion of allyl ether almost fully diminished (as low as 3%, Supplementary Table 4, entry 11). These latter experiments suggest the combined effect of oxygen and CCl_3_CN in promoting the PINO radical from NHPI. Despite the synergistic effect between those reaction parameters in oxidizing the α-C–H bond of allyl ether, a search for terminal oxidant of the process was continued.

Therefore, further investigations were continued with specific oxidants to identify the terminal oxidant. Some reactions were also performed under catalyst-free conditions. The reactivity of oxidant NHPI–air under catalyst-free conditions was found to convert only 12% and converted only 3% when CCl_3_CN and CH_3_CN, the solvent, were excluded (Supplementary Table 4, compare entry 7–12, and 12–14). This further proved the importance of CCl_3_CN as the promoter besides demonstrating that NHPI–air is the nonterminal oxidant. Reactions using air as the sole oxidant were also evaluated. The reaction with air and catalyst converted around 4% of the allyl ether to the monoester and not observed under corresponding catalyst-free conditions (Supplementary Table 4, compare entry 15–16). This confirmed that lattice oxygens of the catalyst were the terminal oxidants.

## Supplementary information

Supplementary Information

## Data Availability

The data that support the findings of this study are available from the corresponding author upon reasonable request.
