# Supplementary material for: Lithium promoted mesoporous manganese oxide catalyzed oxidation of allyl ethers
Source: Nat Commun. 2019 Feb 8;10:655. doi: 10.1038/s41467-019-08619-x (PMC6368603; doi:10.1038/s41467-019-08619-x)
Supplement: Supplementary file 1 — Supplementary Information [file 41467_2019_8619_MOESM1_ESM.docx]

Lithium promoted Mesoporous Manganese Oxide Catalyzed Oxidation of Allyl Ethers

Biswanath Dutta ^1^, Ryan Clarke ^1^, Sumathy Raman ^2^, Timothy D. Shaffer ^2^, Laura Achola^1^

Partha Nandi ^2,^* and Steven L. Suib ^1,3^*

**Affiliations**

*^1^*Department of Chemistry, University of Connecticut, U-3060, 55 North Eagleville Rd., Storrs, Connecticut 06269 (USA), **Fax**: (+1) (860)-486-2981, **E-mail**: [steven.suib@uconn.edu](mailto:steven.suib@uconn.edu)

*^2^* Corporate Strategic Research, ExxonMobil, 1545 US 22 East, Annandale, NJ 08801 (USA) **Email**: partha.nandi@exxonmobil.com.

*^3^*Institute of Materials Science, University of Connecticut, U-3060, 55 North Eagleville Rd., Storrs, Connecticut 06269 (USA).

**SUPPLEMENTARY METHODS**

**Chemicals**

Manganese (II) nitrate tetrahydrate (Mn(NO_3_)_2_.4H_2_O, ≥ 97.0), 1-butanol (anhydrous, 99.8%), and poly (ethylene glycol)- block- Poly (propylene glycol)-block- Poly (ethylene glycol) PEO20-PPO70PEO20 (Pluronic P123), Lithium nitrate, Sodium nitrate, Potassium nitrate, Cesium nitrate, Rubidium nitrate, Calcium nitrate, Barium nitrate and Strontium nitrate were used for preparing different catalysts. For substrate scope Allyl ether, 2,5-Dihydrofuran, allyl acrylate, allyl glycidyl ether, and allyl phenyl ether were used in presence of toluene as solvent, C-Mn_2_O_3_ were purchased from Sigma-Aldrich. Concentrated nitric acid (HNO_3_, 68-70 %) was purchased from J. T. Baker. All chemicals were used as received without further purification.

**Synthesis of Mesoporous Manganese Oxides**

In a typical synthesis 0.02 mol (5.0 g) of manganese nitrate tetrahydrate (Mn (NO_3_)_2_·4H_2_O) and

0.134 mol (10.0 g) of 1-butanol were added into a 120 mL beaker. To this solution 0.00034 mol (2 g) of poly(ethylene glycol)-block-poly(propylene glycol)-block-poly(ethylene glycol) (Pluoronic P123, PEO20PPO70PEO20, molar mass 5750 g mol−1) and 0.032 mol (2 g) of concentrated nitric acid (HNO_3_) were added and stirred at room temperature until the solution became clear (light pink). The resulting clear solution was then kept in an oven at 120°C for 3 h under air. The black product was collected and washed with excess ethanol, centrifuged, and dried in a vacuum oven overnight. At the end, the dried black powders were subjected to a heating cycle. First, they were heated at 150°C for 12 h and cooled down to room temperature under ambient conditions followed by a second heating step of 250°C for 3h.

*For other metal oxides, similar procedure was followed except the calcination cycles.^1^

**Ion promotion of the metal oxides**

After calcination, Metal oxides were taken in a crucible. Onto this 1M of group 1 or group 2 metal ion solutions were drop wise in calculated amounts so that its 1 mol % of the metal oxide taken. After wetness impregnation was done, the crucibles were left for drying and finally they were calcined at 250°C for 30 minutes.

**Preparation of AMO (Amorphous Manganese Oxide)**

The catalyst was synthesized following the procedure described in the literature,[2] by the reduction of KMnO4 with oxalic acid at room temperature. Potassium permanganate solution (1.58 g, 0.01 mol of KMnO4 dissolved in 60 mL of distilled deionized water (DDW)) was added dropwise to the oxalic acid solution (2.28 g, 0.025 mol of oxalic acid dissolved in 100 mL of DDW) and kept stirring for 2 h at room temperature. The brown slurry obtained from the reaction was filtered to get the product. The product was washed several times with deionized water and dried at 90 °C overnight to obtain the amorphous manganese oxide.

**Synthesis of Mesoporous OMS-2**

Amorphous Meso- MnOx (UCT-1) sample (0.3 g) was dispersed in a 50 mL aqueous solution (DDI water) containing 0.5 M H2SO4 and 0.5 M KCl solutions. That mixture was sonicated at room temperature for 10 minutes to form a homogeneous suspension, which was transferred to a glass autoclave and placed in an oven running at 70 oC for 2 h. The obtained powder was filtered and washed several timed with DDI water and finally dried in a vacuum oven over night. The sample is labelled as Meso-OMS-2.

**Synthesis of Mesoporous ɛ-MnOx**

Amorphous Meso-MnOx (UCT-1) sample (0.3 g) was dispersed in 50 mL 0.5M H2SO4 aqueous solution (DDI water) and sonicated at RT for 10 min. That homogeneous suspension was transferred to a glass autoclave and was placed in an oven running at 70 ^o^C for 2 h. The obtained powder was filtered and washed several timed with DDI water and finally dried in a vacuum oven overnight. The sample is labelled as Meso-ɛ-MnOx.

**Experimental procedure**

2 mmol (0.2 g) of allyl ether was taken in a 2 neck 25 mL round bottomed flask, to it 25 mg of catalyst, 2.2 mmol of oxidants and 2.5 mL of the solvent were added successively, in presence of 2 equivalents (0.65 g) of CCl_3_CN and placed in an oil bath at 80°C attaching with a reflux condenser with a septum on top. In the other neck of the reaction vessel another septum was attached. Through this air was bubbled into the solution. Reactions were monitored over the total run time. They were hanged for an hour to cool down. Finally, the mixtures were filtered through syringe filters and analyzed in GC-MS (gas chromatography mass spectrometry). The conversion was determined based on the concentration of allyl ether used. Most reactions were repeated twice to ensure corresponding yields. All products were isolated by silica gel column chromatography (5:95 Ethyl acetate/petroleum ether was used as an eluent).

Characterization

The GC-MS analyses were performed with a 7820A GC system connected with a mass detector of 5975 series MSD from Agilent Technologies and a nonpolar cross-linked methyl siloxane column with dimensions of 12 in × 0.200 mm × 0.33 µm was used. The 1H and 13C NMR spectra were recorded on a Bruker AVANCE III- 400 MHz spectrometer. 1H NMR spectra were collected at 400 MHz with chemical shift referenced to the residual CHCl3 peak in CDCl3 (δ: H 7.26 ppm). 13C NMR spectra were collected at 100 MHz and referenced to the CDCl3 signal (δ: C

77.0 ppm).

**Synthesis of allyl phenyl ethers:**

Allyl bromide and phenol were reacted together for 24 hours at 50-60°C, in presence of potassium carbonate, to produce the corresponding allyl phenyl ether. Potassium carbonates were separated from the crude mixture by solvent extraction procedure. Obtained organic layer was passed through dry MgSO4 to get rid of remaining water. Finally, excess allyl bromides were separated from the product by distillation procedure. Final product was characterized by NMR spectroscopy which was further compared with the literature.

**Synthesis of Amorphous Manganese Oxide (AMO):**

The catalyst was synthesized following the procedure described in the literature,[2] by the reduction of KMnO4 with oxalic acid at room temperature. Potassium permanganate solution (1.58 g, 0.01 mol of KMnO4 dissolved in 60 mL of distilled deionized water (DDW)) was added dropwise to the oxalic acid solution (2.28 g, 0.025 mol of oxalic acid dissolved in 100 mL of DDW) and kept stirring for 2 h at room temperature. The brown slurry obtained from the reaction was filtered to get the product. It was washed several times with deionized water and dried at 90 °C overnight to obtain the amorphous manganese oxide.

**Optimizations:**

**Supplementary Table 1: Solvent: ^a^**

Cl

Cl

Cl

O

O

+

TBHP

N O

I

2 eqv.

| **Entry** | **Solvent (2.5 mL)** | **Conversion (%)** | **Selectivity of I (%)** |
| --- | --- | --- | --- |
| 1. | ACN | 3 | > 99 |
| 2. | CH_2_Cl_2_ | n. d. | n. d. |
| 3. | CHCl_3_ | n. d. | n. d. |
| 4. b | ACN | 19 | > 99 |
| 5. b | CCl_3_CN | 82 | > 99 |
| 6. b | ACN + CCl_3_CN (2 eqv.) | 82 | > 99 |
| 7. b | No solvent | 15 | > 99 |

**^a^ Reaction condition:** Allyl ether (2 mmol), Temp- 80°C, solvent- 2.5 mL, Catalyst- Meso-Mn_2_O_3_ (25mg), ^a^ TBHP (1.1 eqv). 5 hours.^c^ Conversions and selectivities were determined by GC-MS.

**Supplementary Table 2: Metal Oxide: ^a^**

**^a^ Reaction condition:** Allyl ether (2 mmol), Temp- 80°C, ACN- 2.5 mL, CCl_3_CN (2 eqv.), Catalyst- 25 mg, TBHP (1.1 eqv), 5 hours. ^b^ Conversions and selectivities were determined by GC-MS.

| **Entry** | **Catalyst (25mg)** | **Conversion (%) ^b^** | **Selectivity of I (%) ^b^** |
| --- | --- | --- | --- |
| 1. | Meso-MnO_x_ | 82 | > 99 |
| 2. | Meso-CoO_x_ | 3 | > 99 |
| 3. | Meso-CeO_x_ | 3 | > 99 |
| 4. | Meso-ZrO_x_ | 3 | > 99 |
| 5. | AMO | 19 | > 99 |
| 6. | c-Mn_2_O_3_ | 20 | > 99 |
| 7. | Mn(OAc)_3_ | 13 | > 99 |
| 8. | Mn(OAc)_2_ | 6 | > 99 |
| 9. | No catalyst | 3 | > 99 |
| 10. | Meso-OMS-2 | 42 | > 99 |
| 11. | Meso-ε-MnOx | 35 | > 99 |

**Supplementary Table 3: Ion-Impregnation: ^a^**

| **Entry** | **Catalyst (25mg)** | **Conversion (%) ^b^** | **Selectivity of I (%) ^b^** |
| --- | --- | --- | --- |
| 1. | Meso-MnO_x_ | 82 | > 99 |
| 2. | Meso-CoO_x_ | 3 | > 99 |
| 3. | Meso-CeO_x_ | 3 | > 99 |
| 4. | Meso-ZrO_x_ | 3 | > 99 |
| 5. | AMO | 19 | > 99 |
| 6. | c-Mn_2_O_3_ | 20 | > 99 |
| 7. | Mn(OAc)_3_ | 13 | > 99 |
| 8. | Mn(OAc)_2_ | 6 | > 99 |
| 9. | No catalyst | 3 | > 99 |
| 10. | Meso-OMS-2 | 42 | > 99 |
| 11. | Meso-ε-MnOx | 35 | > 99 |

**a**

**Reaction condition:** Allyl ether (2 mmol), Temp- 80°C, ACN- 2.5 mL, CCl_3_CN (2 eqv.), Catalyst- (25mg), TBHP (1.1 eqv), 5 hours. ^b^ Conversions and selectivities were determined by GC-MS.

**Supplementary Table 4: Oxidant: ^a^**

| **Entry** | **Oxidant** | **Conversion (%) ^f^** | **Selectivity of I (%)^f^** |
| --- | --- | --- | --- |
| 1. | TBHP | 92 | > 99 |
| 2. | H2O2 | 10 | 40 |
| 3. | m-CPBA | 30 | > 99 |
| 4. | Cumin Peroxide | 55 | > 99 |
| 5. | Benzoyl peroxide | 90 | > 99 |
| 6. | Peracetic acid | 90 | 63 |
| 7. | **NHPI-air** | **95** | **> 99** |
| 8. b | **NHPI-air** | **60** | **> 99** |
| 9. c | **NHPI-air** | **80** | **> 99** |
| 10. ^b^ | **NHPI-N_2_** | **18** | **> 99** |
| 11. b, c | **NHPI-N_2_** | **3** | **> 99** |
| 12. ^d^ | **NHPI-air** | **12** | **> 99** |
| 13. ^e^ | **NHPI-air** | **24** | **> 99** |
| 14. d, e | **NHPI-air** | **6** | **> 99** |
| 15. ^d^ | Air | n.d | n.d. |
| 16. | Air | **4** | **> 99** |

**^a^ Reaction condition:** Allyl ether (2 mmol), Temp- 80°C, ACN- 2.5 mL, CCl_3_CN (2 eqv.), Catalyst- Meso-Li-Mn_2_O_3_ (25 mg), Oxidant (1.1 eqv.), 5 hours. ^b^ 2 hours. ^c^ in absence of CCl_3_CN. ^d^ Without Catalyst_,_ ^e^ solvent free. ^f^ Conversions and selectivities were determined by GC-MS.

**Supplementary Table 5: NHPI loading:** ^a^

| **Entry** | **NHPI loading (mol %)** |  | **Conversion (%)^b^** | **Selectivity of I**  **(%)^b^** |
| --- | --- | --- | --- | --- |
| 1. | 1 |  | 28 | > 99 |
| 2. | 5 |  | 42 | > 99 |
| 3. | 10 |  | 53 | > 99 |
| 4. | 25 |  | 86 | > 99 |
| 5. | 50 |  | 90 | > 99 |
| 6. | 100 |  | 92 | > 99 |
| 7. | 110 |  | 95 | > 99 |

**^a^ Reaction condition:** Allyl ether (2 mmol), Temp- 80°C, ACN- 2.5 mL, CCl_3_CN (2 eqv.), Catalyst- Meso-Li-Mn_2_O_3_ (25 mg), 5 hours, **air balloon**. ^b^ Conversions and selectivities were determined by GC-MS.


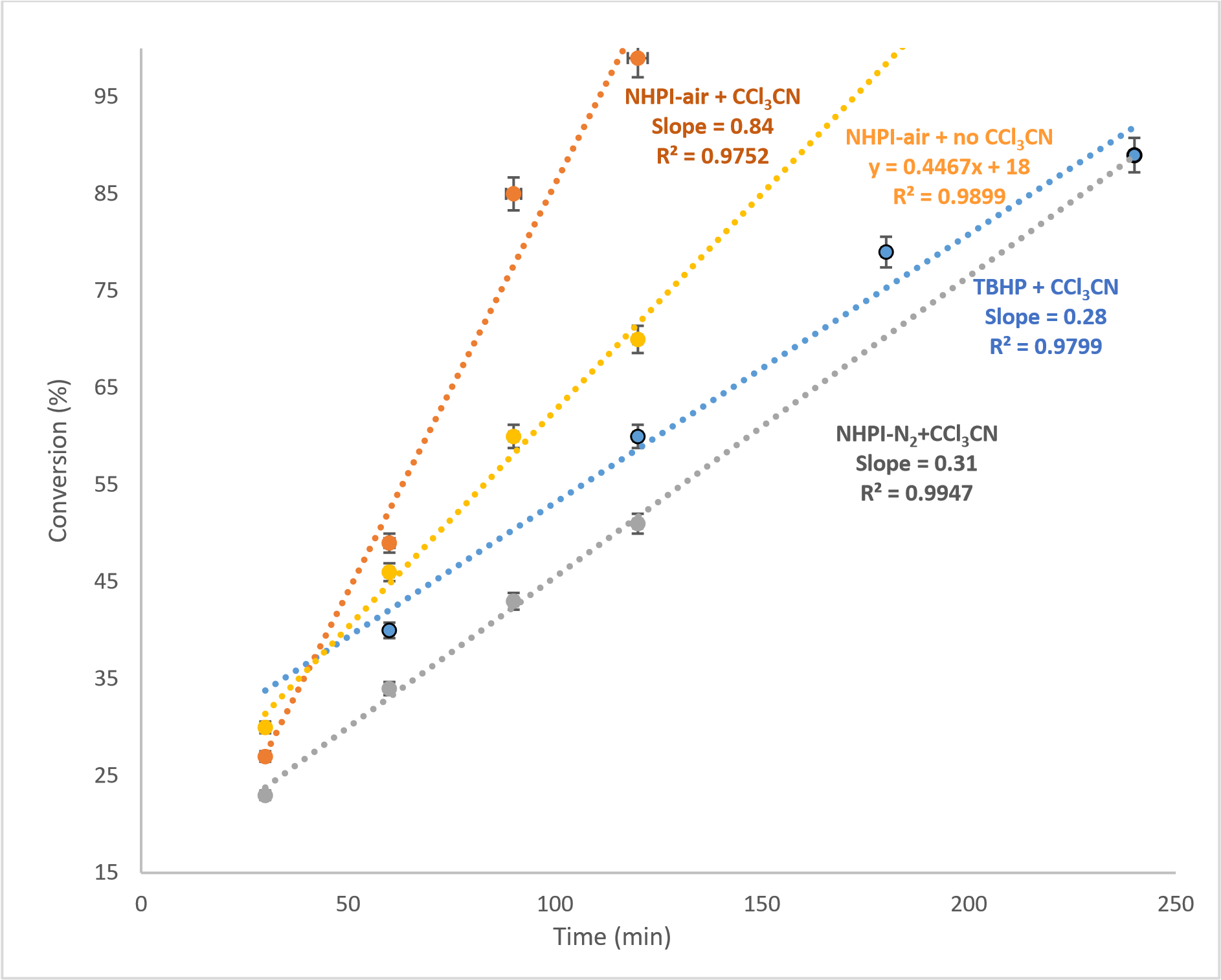


**Supplementary Figure 1: Reaction Condition**: Allyl ether (0.5 mmol), Solvent (2.5 mL), Oxidant (25 mol%), 25 mg of Meso-Li-Mn_2_O_3_ at 80°C.


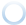

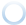

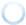

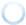

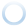

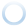

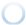

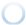


**TBHP + CCl3CN**

**Slope =**

**-0.7113**

**R² = 0.9943**

**NHPI-air + CCl3CN**

**Slope =-2.819**

**R² = 0.8765**

**NHPI-N2 + CCl3CN**

**Slope = -0.3005**

**R² = 0.9999**

**NHPI-air +No CCl3CN**

**Slope = -0.5684**

**R² = 0.9992**

-5

-4.5

-4

-3.5

-3

-2.5

-2

-1.5

-1

-0.5

0

0.5

1

1.5

2

2.5

3

3.5

4

4.5

ln (A

t

/A

0

)

Time (h)

**Supplementary Figure 2: Reaction Condition**: Allyl ether (0.5 mmol), Solvent (2.5 mL), oxidant (25 mol %), 25 mg of Meso-Li-Mn_2_O_3_ at 20°C.

**Supplementary Table 6: NHPI loading: ^a^**

| **Entry** | **Reaction Initiator** | **Reaction Environment** | **CCl_3_CN** | **Rate Constant (min^-1^)** |
| --- | --- | --- | --- | --- |
| 1.^b^ | NHPI | **N_2_** | Yes | 0.30 |
| 2. | NHPI | air | **No** | 0.57 |
| 3. | **TBHP** | air | Yes | 0.71 |
| **4.** | **NHPI** | **air** | **Yes** | **2.82** |

**^a^ Reaction condition:** Allyl ether (0.5 mmol), Temp- 80°C, ACN- 2.5 mL, CCl_3_CN (2 eqv.), Catalyst- Meso-Li-Mn_2_O_3_ (25mg), Oxidant (1.1 eqv.), 5 hours, **air balloon**. ^b^ The solvent was not thoroughly degassed to get rid of dissolved O_2_ prior to the run.

0

10

20

30

40

50

60

70

80

90

100

-50

0

50

100

150

200

250

300

350

400

Conversion (%)

Time(minutes)

**Supplementary Figure 3:** Effect of removal of catalyst on the oxidation of **allyl ethers** (0.5 mmol), Solvent (2.5 mL), oxidant (25 mol %), 25 mg Meso-Li-Mn_2_O_3_ at 20°C. After **45 minutes**, catalyst was removed by hot filtration (at about **25** % conversion). GC-MS. determined conversions

0

5

10

15

20

25

30

Cycle 1

Cycle 2

Cycle 3

Cycle 4

**Conversion and Yield (%)**

**Number of cycles**

Reusability

Selectivity (%)

Yield (%)

**Supplementary Figure 4.** Reusability test of the catalyst. Reaction condition: **allyl ethers** (2 mmol), Solvent (2.5 mL), oxidant (25 mol %), 25 mg of Meso-Li-Mn_2_O_3_ at 20°C, 30 minutes.

Conversions and selectivities were determined by GC-MS.

a)

**2000**

**3000**

**4000**

**5000**

**-0.3**

**-0.2**

**-0.1**

**0.0**

**0.1**

**0.2**

**0.3**

**meso-**

**MnOx + NHPI**

**MnOx**

**meso-**

**Field (G)**

**Intensity(a. u.)**

**b)**

**2800**

**3500**

**4200**

**-1**

**0**

**1**

**Intensity (a. u.)**

**G**

**NHPI + Mn(acac)**

**3**

**Mn(acac)**

_3_

**Supplementary Figure 5.** EPR of a) comparison between meso-Mn_2_O_3_ and meso-Mn_2_O_3_ with NHPI. b) comparison between Mn(acac)_3_ and Mn(acac)_3_ with NHPI

**a)**

**b)**

**c)**


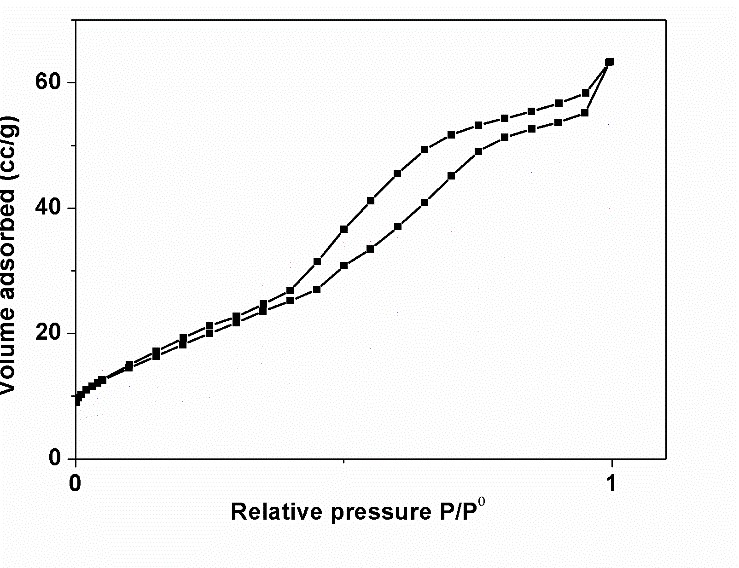

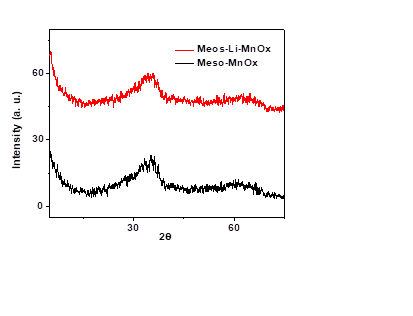

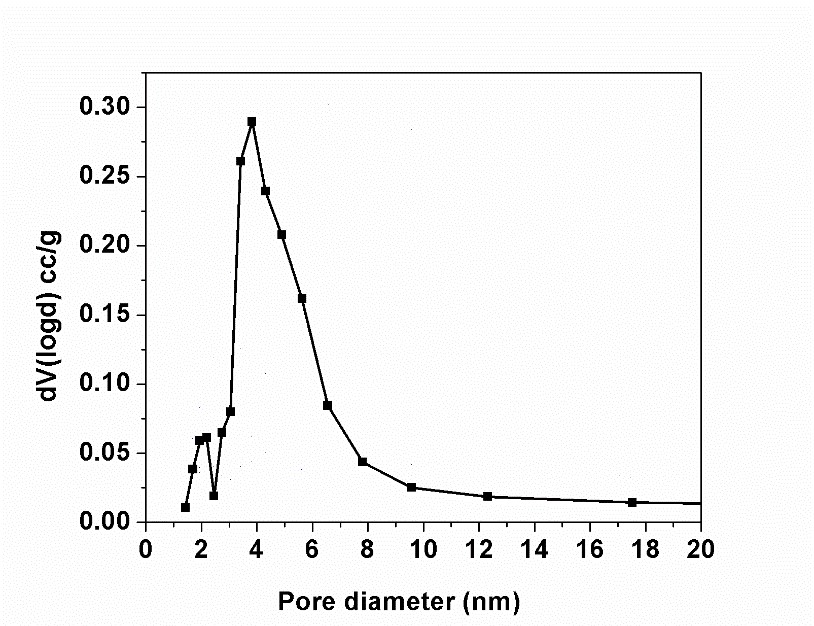


**Supplementary Figure 6.** a) X-ray diffraction pattern, b) BET isotherm, and c) BJH pore size distribution of meso-MnOx material.

**Supplementary Note 1**

**The following are NMR data of various species in this work.**

## Allyl Acrylate

**^1^H NMR (300 MHz, CDCl_3_):** ∂ 6.48 – 6.40 (dd, 1 H), 5.85 – 5.82 (dd, 1 H), 5.39 – 5.32 (q, 2 H), 4.69 – 4.65 (d, 2 H), 6.20 – 6.10 (m, 1 H), 6.01 – 5.86 (m, 1 H), ppm.

* = impurities


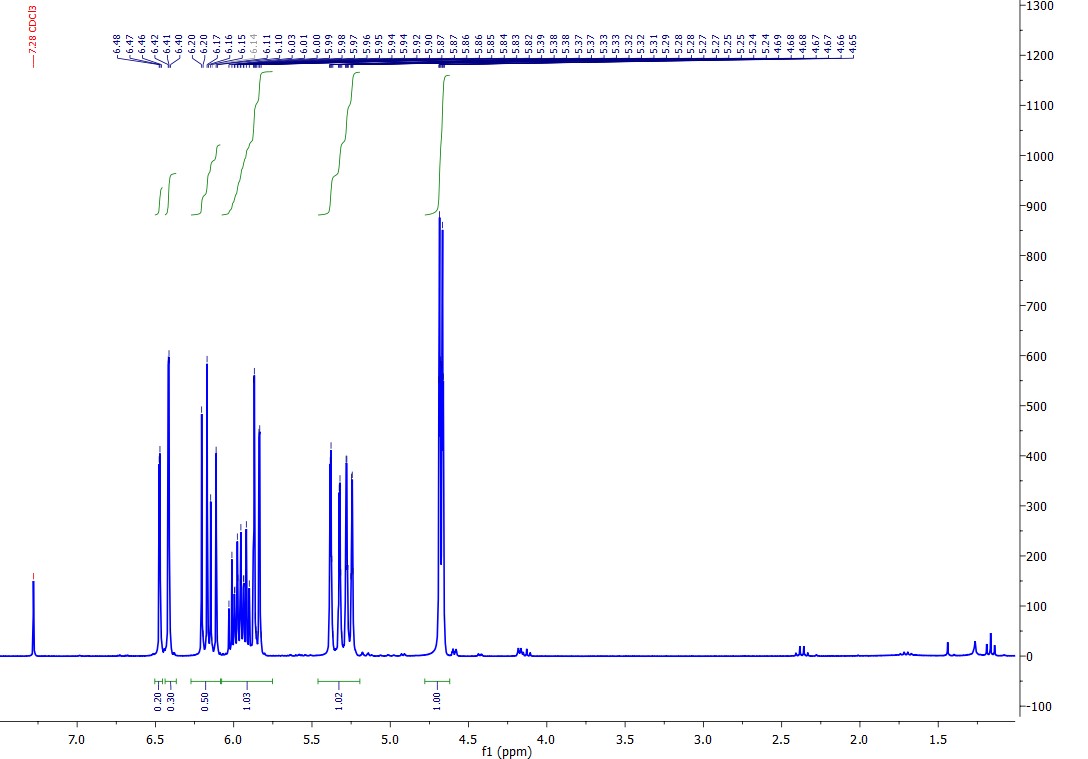

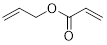

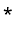

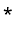

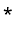

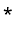

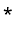

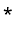


∂ 165.8, 132.0, 130.9, 128.3, 118.3, 65.2 ppm.


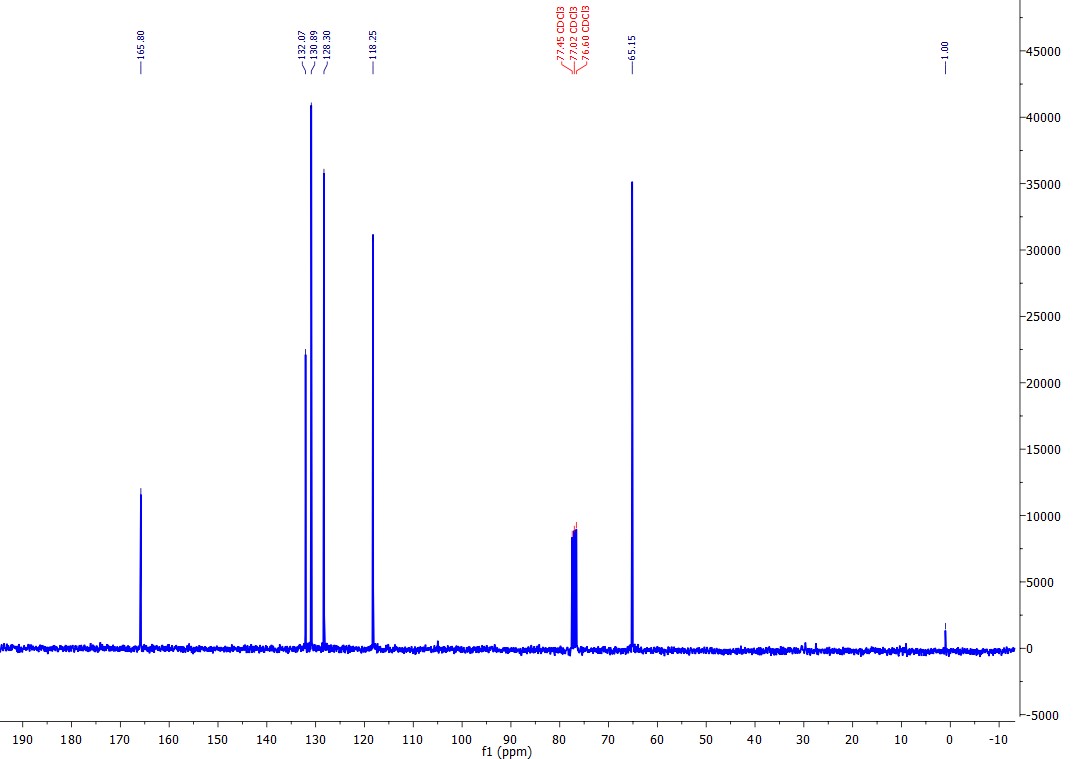


## 2(5*H*)-Furanone

**^1^ H NMR (300 MHz, CDCl3):** ∂ 7.61 – 7.57 (m, 1 H), 6.18 – 6.13 (m, 1 H), 4.92 – 4.88 (m, 2 H), ppm.


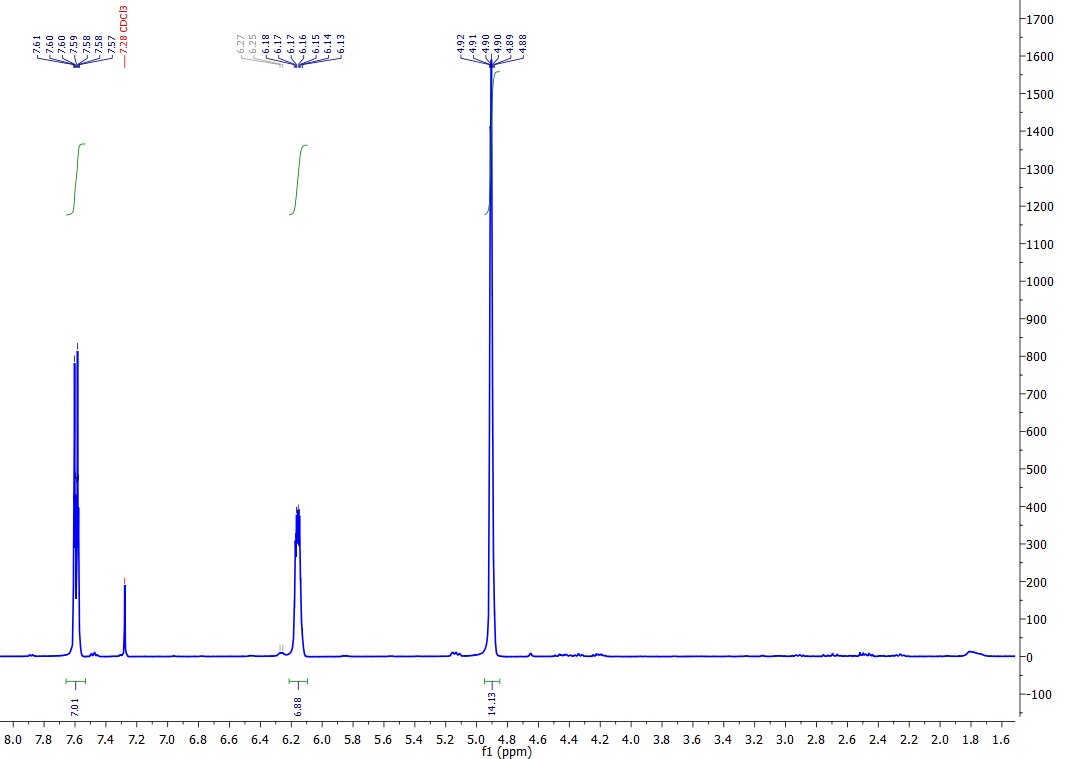

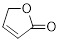


∂ 177.7, 152.9, 121.5, 72.2 ppm.


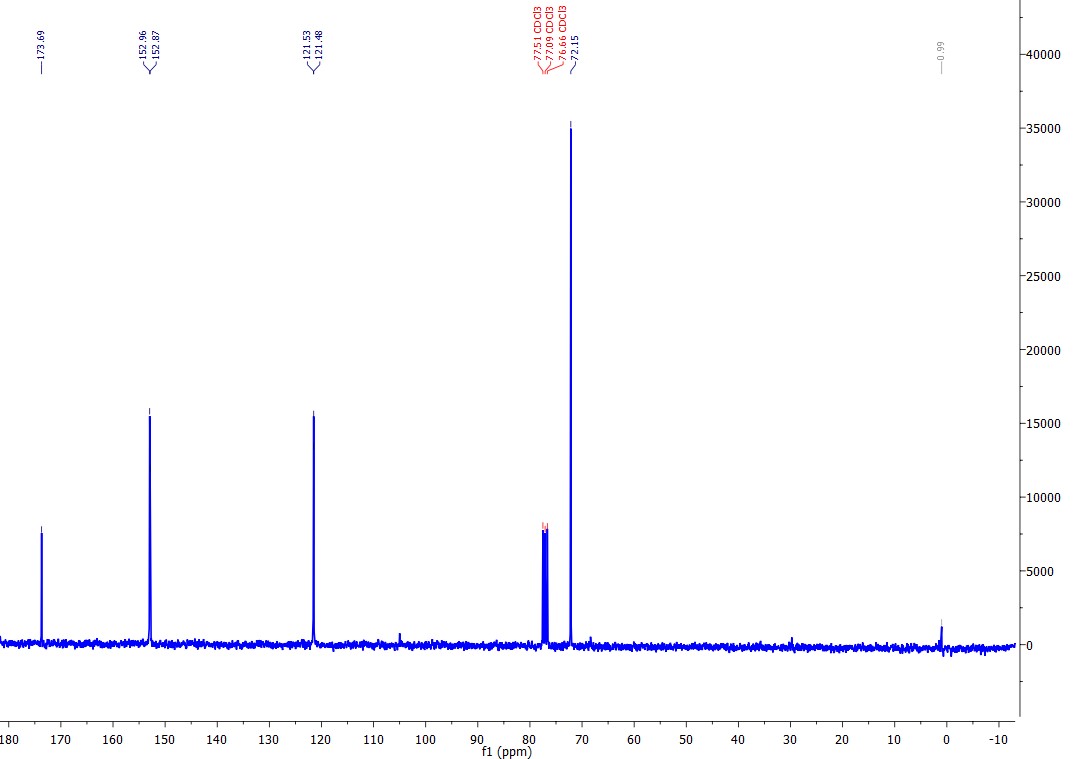


## Glycidyl Acrylate

**^1^H NMR (300 MHz, CDCl_3_):** ∂ 6.50 – 6.44 (m, 1 H), 6.22 – 6.13 (m, 1 H), 5.91 – 5.88 (m, 1 H), 4.53 – 4.0 (m, 2 H), 3.29 – 3.24 (m, 1 H), 2.89 – 2.67 (m, 2 H) ppm.


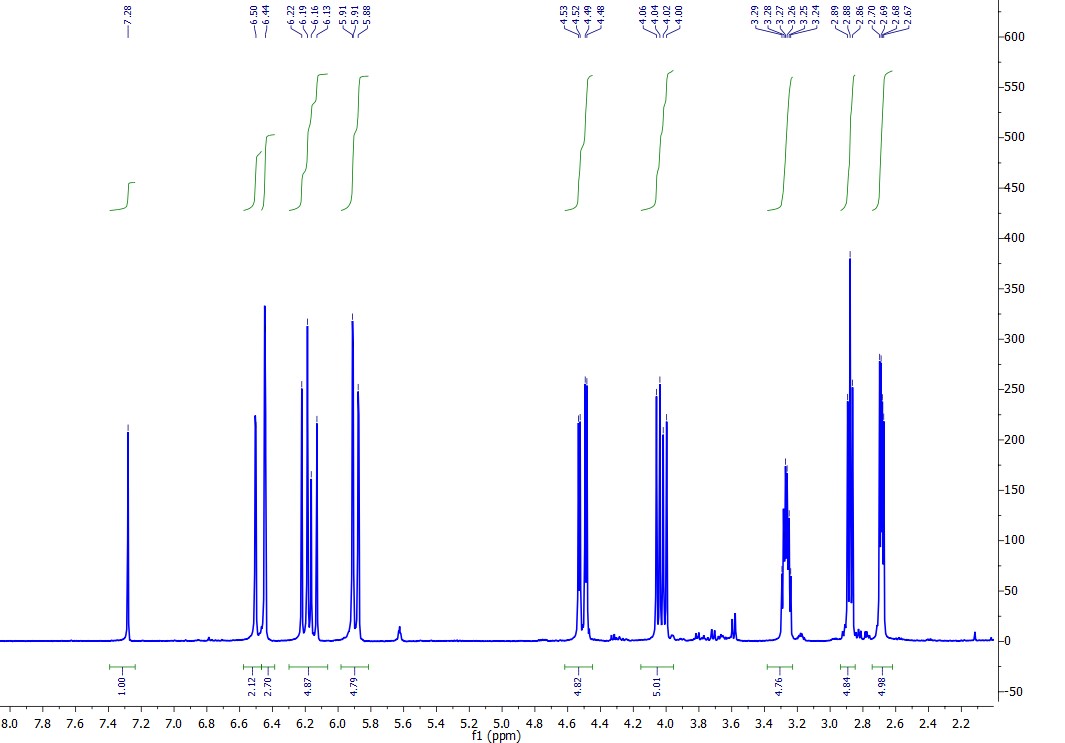

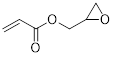


∂ 165.8, 131.5, 127.9, 126.2, 65.0, 49.3, 44.7 ppm.


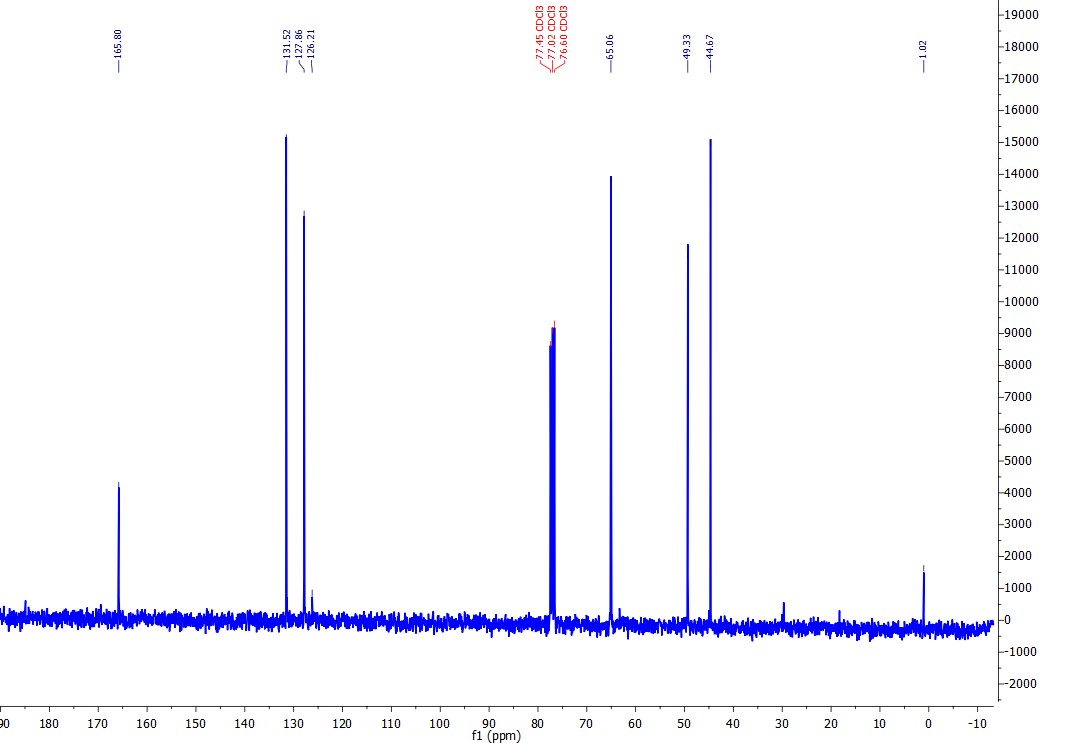


## 2-propionic acid-phenyl ester

Abundance


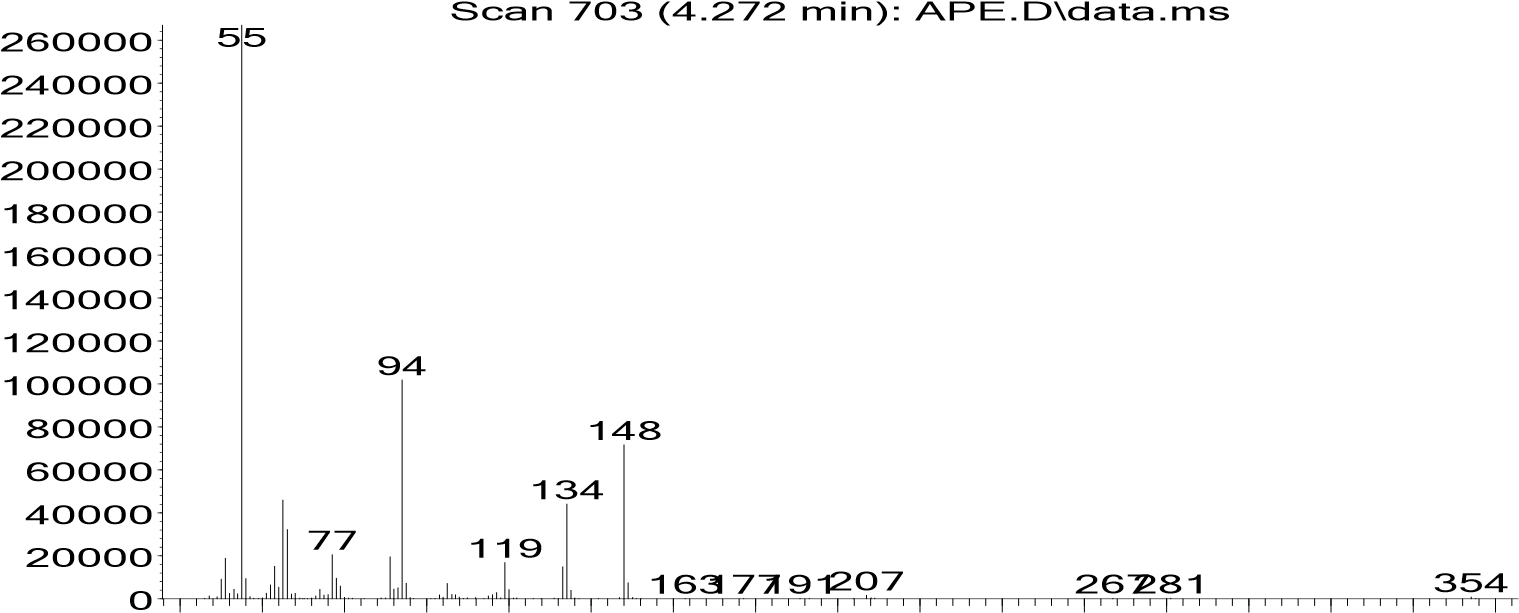


40 60 80 100120140160180200220240260280300320340

m/z-->

replib) 2-Propenoic acid, phenyl ester

(

40

80

120

160

200

240

280

320

360

400

440

0

50

100

55

65

77

94

120

148

O

O
